# Supplementary material for: The COVID-19 Social Monitor longitudinal online panel: Real-time monitoring of social and public health consequences of the COVID-19 emergency in Switzerland
Source: PLoS One. 2020 Nov 11;15(11):e0242129. doi: 10.1371/journal.pone.0242129 (PMC7657546; doi:10.1371/journal.pone.0242129)
Supplement: S1 Table — (DOCX) [file pone.0242129.s001.docx]

**S1 Table:** Scheme for follow-up survey waves (planned, depending on pandemic/public health measures dynamic and financing).

| Week Number  (start March 30, 2020) | 14 | 15 | 16 | 18 | 20 | 22 | 25 | 29 | 34 | 40 | ... |
| --- | --- | --- | --- | --- | --- | --- | --- | --- | --- | --- | --- |
| Waves of survey | 1 | 2 | 3 | 4 | 5 | 6 | 7 | 8 | 9 | 10 | ... |

Stepwise (expected/announced) relaxation of lockdown measures in Switzerland: End of weeks 16, 20 and 23.
