# Supplementary material for: The COVID-19 Social Monitor longitudinal online panel: Real-time monitoring of social and public health consequences of the COVID-19 emergency in Switzerland
Source: PLoS One. 2020 Nov 11;15(11):e0242129. doi: 10.1371/journal.pone.0242129 (PMC7657546; doi:10.1371/journal.pone.0242129)
Supplement: S2 Table — (DOCX) [file pone.0242129.s002.docx]

**S2 Table:** Table of items used for the first survey wave questionnaire^*^.

| **Domain** | **Source** | **Original survey question (only German)** |
| --- | --- | --- |
| Subjective health | SHS | Wie ist Ihr Gesundheitszustand im Allgemeinen? |
| Health problems (last 7d) | SHS adapted | Hatten Sie in den letzten 7 Tagen eines der folgenden Symptome? |
| Perceived stress (last 7d) | SHP | Wie oft haben Sie Stress empfunden während der letzten 7 Tage? |
| Mental Health Score (MOS (last 7d)) | SHS | Wie haben Sie sich in den letzten 7 Tagen gefühlt? |
| General life satisfaction (0-10) | SHP | Ganz allgemein gefragt - wie zufrieden sind Sie mit Ihrem Leben? 0 "gar nicht zufrieden' und 10 "vollumfänglich zufrieden'. |
| Quality of life | SHS | Wie schätzen Sie Ihre Lebensqualität im Allgemeinen im Moment ein? |
| Quality of life change due to COVID | new | Wie ist ihre Lebensqualität im Moment im Vergleich zur Zeit vor der Corona-Krise? |
| Leaving home per day 15 min (last 7d) | new | In den letzten 7 Tagen: Wie viel Mal am Tag haben Sie Ihr Zuhause durchschnittlich für mind. 15 Minuten verlassen? |
| Reason leaving home 15min (last 7d) | www.sotomo.ch, adapted | Wofür haben Sie ihr Zuhause in den letzten 7 Tagen verlassen? (mehrere Antworten möglich) |
| Physical activity: days (last 7d) | Wanner et al. 2014,  doi: 10.1016/j.ypmed.2014.01.025 | An wie vielen Tagen der letzten Woche waren Sie insgesamt 30 Minuten oder länger körperlich aktiv, so dass Sie zumindest etwas stärker atmen mussten? Beispiele für solche Aktivitäten sind Sport, Bewegung, Training sowie zügiges Gehen oder Velofahren, entweder in der Freizeit oder um von Ort zu Ort zu gelangen. Körperliche Aktivitäten im Haushalt oder im Rahmen Ihrer Arbeit berücksichtigen Sie hingegen bitte nicht. |
| Living with partner | new | Haben Sie einen (Ehe-)Partner oder eine (Ehe-)Partnerin? |
| Household co-living satisfaction | SHP | Wie zufrieden sind Sie mit dem Zusammenleben in Ihrem Haushalt? |
| Partner relationship satisfaction | SHP | Wie glücklich sind Sie im Allgemeinen in Ihrer aktuellen Partnerschaft? |
| Loneliness | SHS | Wie häufig kommt es momentan vor, dass Sie sich einsam fühlen? |
| Working status | SHS adapted | Haben Sie derzeit eine bezahlte Arbeit? (egal ob angestellt oder selbstständig) |
| Regular working hours | new | Wie viele Stunden haben Sie in einer normalen Arbeitswoche vor der Corona-Krise gearbeitet? |
|  |  |  |
|  |  |  |
| Hours lost due to crisis (excl. illness, last 7d) | WPAI adapted | Wie viele Arbeitsstunden haben Sie in den letzten 7 Tagen wegen der Corona-Krise versäumt? (z.B. wegen Vermeidung von Ansteckung, Schliessung des Geschäfts, Betreuung von Kindern infolge Schulschliessungen – aber ohne allfällige eigene Corona-Infektion) |
| Hours lost due to illness (incl. COVID, last 7d) | WPAI adapted | Wie viele Arbeitsstunden haben Sie in den letzten 7 Tagen wegen Krankheit oder Unfall versäumt? (inklusive wegen eigener Corona-Infektion) |
| Hours lost due to other reasons (last 7d) | WPAI adapted | Wie viele Arbeitsstunden haben Sie in den letzten 7 Tagen aus anderen Gründen versäumt, wie z.B. Ferien oder Feiertage? |
| Hours actually worked (last 7d) | WPAI adapted | Wie viele Stunden haben Sie in den letzten 7 Tagen tatsächlich gearbeitet? (egal ob am üblichen Arbeitsort oder im Homeoffice) |
| Productivity loss work (last 7d) | WPAI adapted | Wie stark hat sich die Corona-Krise in den letzten 7 Tagen auf Ihre Produktivität bei der Arbeit ausgewirkt? |
|  |  | Denken Sie an Tage, an denen Sie hinsichtlich der Menge oder Art der Arbeit, die Sie erledigen konnten, eingeschränkt waren, an denen Sie weniger bewältigten als Sie wollten, oder an denen Sie Ihre Arbeit nicht so sorgfältig wie üblich erledigen konnten. |
| Productivity loss activities of daily living (last 7d) | WPAI adapted | Wie stark hat sich die Corona-Krise in den letzten 7 Tagen auf Ihre alltäglichen Beschäftigungen ausserhalb der Berufstätigkeit ausgewirkt? |
|  |  | (z.B. Hausarbeit, Einkaufen, Kinderbetreuung, körperliches Training, Studieren) |
|  |  | Denken Sie an Zeiten, in denen Sie hinsichtlich der Menge oder Art der Aktivitäten, die Sie erledigen konnten, eingeschränkt waren, und in denen Sie weniger bewältigten, als Sie wollten. |
| Short-time working | new | Leisteten Sie in den letzten 7 Tagen Kurzarbeit? |
| Fear of losing job | SHS | Haben Sie Angst, Ihren heutigen Arbeitsplatz zu verlieren? |
| Homeoffice due to COVID (last 7 days) | new | Haben Sie in den letzten 7 Tagen von zuhause aus gearbeitet (Homeoffice)? |
| Homeoffice prior to COVID | new | Arbeiteten Sie schon vor der Corona-Krise von zu Hause aus? |
| Health services use (last 14 days, from 2nd wave on 7 days) | new, partly based on SHS list | Haben Sie in den letzten 14 Tagen eine medizinische Behandlung erhalten? |
| Health service non-use (last 14 days, from 2nd wave on 7 days) | new, partly based on SHS list | Konnten Sie in den letzten 14 Tagen eine geplante oder nötige medizinische Behandlung nicht in Anspruch nehmen wegen der Corona-Krise? |
|  |  | z.B. weil der Termin durch den Arzt/Spital abgesagt oder verschoben wurde oder weil Sie aktuell kein Spital/Arztpraxis aufsuchen möchten |
|  |  | (Mehrere Antworten möglich) |
| Health service non-use: type | new | Handelte es sich bei der Behandlung, die Sie nicht beanspruchen konnten wegen der Corona-Krise, um… |
| Health service non-use: reason | new | Was war der Grund, wieso Sie diese Behandlung nicht beanspruchen konnten? |
| Health service use due to COVID (last 14 days) | new | Haben Sie in den letzen 7 Tagen einen Arzt oder ein Spital kontaktiert wegen einem Gesundheitsproblem von Ihnen, das mit dem Corona-Virus zu tun hatte? |

* Questions are shown in the primary language German. Translations in French and Italian not shown.

Abbreviations: SHS Swiss Health Survey; SHP Swiss Household Panel; WPAI Work Productivity and Activity Impairment Questionnaire.
