# Supplementary material for: The COVID-19 Social Monitor longitudinal online panel: Real-time monitoring of social and public health consequences of the COVID-19 emergency in Switzerland
Source: PLoS One. 2020 Nov 11;15(11):e0242129. doi: 10.1371/journal.pone.0242129 (PMC7657546; doi:10.1371/journal.pone.0242129)
Supplement: S3 Table — (DOCX) [file pone.0242129.s003.docx]

**S3 Table:** Post-stratification weighted study outcome results of N=2,026 survey participants.

| Study outcome | n | Percentage | Lower 95% CI | Upper 95% CI |
| --- | --- | --- | --- | --- |
| **General health and well-being** |  |  |  |  |
| General life satisfaction: Good to very good | 2,025 | 94.0% | 92.8% | 95.0% |
| Current quality of life: Good to very good | 2,026 | 85.5% | 83.6% | 87.1% |
| Quality of life compared to before COVID-19 emergency: Worsened | 2,025 | 41.0% | 38.6% | 43.5% |
| General health status: Good to very good | 2,025 | 87.8% | 86.0% | 89.4% |
| **Social well-being** |  |  |  |  |
| Feelings of loneliness: Often or very often | 2,026 | 9.1% | 7.8% | 10.5% |
| **Physical activity** |  |  |  |  |
| No moderate physical activity during the last 7 days | 2,025 | 18.6% | 16.7% | 20.6% |
| Never left home during the last 7 days | 2,025 | 4.1% | 3.1% | 5.4% |
| **Health service use** |  |  |  |  |
| Medical treatment received (last 14 days) | 2,025 | 16.8% | 14.9% | 18.8% |
| Non-use of medical treatment (last 14 days) | 2,026 | 22.3% | 20.2% | 24.5% |
| **Working situation** |  |  |  |  |
| Unemployed due to Corona-Crisis | 1,494^*^ | 1.4% | 0.9% | 2.3% |
| Already unemployed before Corona-Crisis | 1,494^*^ | 2.3% | 1.6% | 3.3% |
| Fears of losing employment** | 1,435^*^ | 10.1% | 8.5% | 11.9% |
| Home office during the last 7 days** | 1,435^*^ | 53.2% | 50.3% | 56.0% |
| Home office before Corona-Crisis** | 1,435^*^ | 26.4% | 24.0% | 29.0% |

Abbreviations: CI Confidence interval.

^*^ Denominator: Employed population (N=1,494).
