# Supplementary material for: The COVID-19 Social Monitor longitudinal online panel: Real-time monitoring of social and public health consequences of the COVID-19 emergency in Switzerland
Source: PLoS One. 2020 Nov 11;15(11):e0242129. doi: 10.1371/journal.pone.0242129 (PMC7657546; doi:10.1371/journal.pone.0242129)
Supplement: S1 File — (PDF) [file pone.0242129.s004.pdf]

# COVID-19 social monitor: Table 1 and Table 2

Analysis code for Table 1 and Table 2 of "The COVID-19 social monitor longitudinal online panel: Real-time monitoring of social and public health consequences of the COVID-19 emergency in Switzerland".

```
library(tidyverse)
library(xtable)
library(tableone)

data <- read_csv(paste0(main.path, "data.csv"))

n_col <- ncol(data)+1

# General life satisfaction
data$wb1_cat <- ifelse(data$wb1>=7, 1, 0)
# Quality of life
data$wb2_cat1 <- ifelse(data$wb2<=2, 1, 0)
# Quality of life before COVID-19 emergency
data$wb3_cat3 <- ifelse(data$wb3<=2, 1, 0)
# Subjective health status
data$ph1_cat1 <- ifelse(data$ph1<=2, 1, 0)
# Disease symptoms
data$ph2_1_cat1 <- ifelse(data$ph2_1==3, 1, 0)
data$ph2_2_cat1 <- ifelse(data$ph2_2==3, 1, 0)
data$ph2_3_cat1 <- ifelse(data$ph2_3==3, 1, 0)
data$ph2_4_cat1 <- ifelse(data$ph2_4==3, 1, 0)
data$ph2_5_cat1 <- ifelse(data$ph2_5==3, 1, 0)
data$ph2_6_cat1 <- ifelse(data$ph2_6==3, 1, 0)
data$ph2_7_cat1 <- ifelse(data$ph2_7==3, 1, 0)
data$ph2_8_cat1 <- ifelse(data$ph2_8==3, 1, 0)
data$ph2_9_cat1 <- ifelse(data$ph2_9==3, 1, 0)
data$ph2_10_cat1 <- ifelse(data$ph2_10==3, 1, 0)
data$ph2_11_cat1 <- ifelse(data$ph2_11==3, 1, 0)
data$ph2_12_cat1 <- ifelse(data$ph2_12==3, 1, 0)
data$ph2_1_cat3 <- ifelse(data$ph2_1==1, 1, 0)
data$ph2_2_cat3 <- ifelse(data$ph2_2==1, 1, 0)
data$ph2_3_cat3 <- ifelse(data$ph2_3==1, 1, 0)
data$ph2_4_cat3 <- ifelse(data$ph2_4==1, 1, 0)
data$ph2_5_cat3 <- ifelse(data$ph2_5==1, 1, 0)
data$ph2_6_cat3 <- ifelse(data$ph2_6==1, 1, 0)
data$ph2_7_cat3 <- ifelse(data$ph2_7==1, 1, 0)
data$ph2_8_cat3 <- ifelse(data$ph2_8==1, 1, 0)
data$ph2_9_cat3 <- ifelse(data$ph2_9==1, 1, 0)
data$ph2_10_cat3 <- ifelse(data$ph2_10==1, 1, 0)
data$ph2_11_cat3 <- ifelse(data$ph2_11==1, 1, 0)
data$ph2_12_cat3 <- ifelse(data$ph2_12==1, 1, 0)
data$ph2_1_cat2 <- ifelse(data$ph2_1==2, 1, 0)
data$ph2_2_cat2 <- ifelse(data$ph2_2==2, 1, 0)
data$ph2_3_cat2 <- ifelse(data$ph2_3==2, 1, 0)
```

```r
data$ph2_4_cat2 <- ifelse(data$ph2_4==2, 1, 0)
data$ph2_5_cat2 <- ifelse(data$ph2_5==2, 1, 0)
data$ph2_6_cat2 <- ifelse(data$ph2_6==2, 1, 0)
data$ph2_7_cat2 <- ifelse(data$ph2_7==2, 1, 0)
data$ph2_8_cat2 <- ifelse(data$ph2_8==2, 1, 0)
data$ph2_9_cat2 <- ifelse(data$ph2_9==2, 1, 0)
data$ph2_10_cat2 <- ifelse(data$ph2_10==2, 1, 0)
data$ph2_11_cat2 <- ifelse(data$ph2_11==2, 1, 0)
data$ph2_12_cat2 <- ifelse(data$ph2_12==2, 1, 0)
# Mental problems
data$ms2_1_cat1 <- ifelse(data$ms2_1<=2, 1, 0)
data$ms2_1_cat3 <- ifelse(data$ms2_1==5, 1, 0)
data$ms2_1_cat2 <- ifelse(data$ms2_1<5 & data$ms2_1>2, 1, 0)
data$ms2_2_cat1 <- ifelse(data$ms2_2<=2, 1, 0)
data$ms2_2_cat3 <- ifelse(data$ms2_2==5, 1, 0)
data$ms2_2_cat2 <- ifelse(data$ms2_2<5 & data$ms2_2>2, 1, 0)
data$ms2_3_cat1 <- ifelse(data$ms2_3>=4, 1, 0)
data$ms2_3_cat3 <- ifelse(data$ms2_3==1, 1, 0)
data$ms2_3_cat2 <- ifelse(data$ms2_3<4 & data$ms2_3>1, 1, 0)
data$ms2_4_cat1 <- ifelse(data$ms2_4<=2, 1, 0)
data$ms2_4_cat3 <- ifelse(data$ms2_4==5, 1, 0)
data$ms2_4_cat2 <- ifelse(data$ms2_4<5 & data$ms2_4>2, 1, 0)
data$ms2_5_cat1 <- ifelse(data$ms2_5>=4, 1, 0)
data$ms2_5_cat3 <- ifelse(data$ms2_5==1, 1, 0)
data$ms2_5_cat2 <- ifelse(data$ms2_5<4 & data$ms2_5>1, 1, 0)
data$ms2_6_cat1 <- ifelse(data$ms2_6>=4, 1, 0)
data$ms2_6_cat3 <- ifelse(data$ms2_6==1, 1, 0)
data$ms2_6_cat2 <- ifelse(data$ms2_6<4 & data$ms2_6>1, 1, 0)
data$ms2_7_cat1 <- ifelse(data$ms2_7<=2, 1, 0)
data$ms2_7_cat3 <- ifelse(data$ms2_7==5, 1, 0)
data$ms2_7_cat2 <- ifelse(data$ms2_7<5 & data$ms2_7>2, 1, 0)
# Feelings of isolation
data$ss3_catx <- ifelse(data$ss3>=4, 1, 0)
# Health service use
data$hs1_1_cat <- ifelse(data$hs1_1==1, 0, 1)
# Health service non-use
data$hs2_1_cat <- ifelse(data$hs2_1==1, 0, 1)
# Fears losing employment
data$wc1_cat <- ifelse(data$wc1<=2, 1, 0)
# Homeoffice last 7 days
data$wc2_cat <- ifelse(data$wc2<=3, 1, 0)
# Never left home
data$hb1_cat <- ifelse(data$hb1==0, 1, 0)
# No physical activity
data$hb7_cat <- ifelse(data$hb7==0, 1, 0)

names_var <- names(data)[n_col:ncol(data)]
names_var <- c(names_var, "unempl_prior", "unempl_covid")
ind <- which(names(data)%in%names_var)

output <- c()
for (i in 1:length(ind)) {
  mod <- glm(c(data[,ind[i] ])[[1]] ~ 1, family=binomial())
```

```
  output2 <- data.frame(var=names(data)[ind[i]],
                        val=plogis(mod$coefficients),
                        lci=plogis(confint.default(mod))[1],
                        uci=plogis(confint.default(mod))[2],
                        n=mod$df.null+1)

  output <- rbind(output, output2)
  output <- as_tibble(output)
}
```

```
data$lregion <- factor(data$lregion, levels=1:3,
                       labels=c("German/Romansh", "French", "Italian"))
data$agecat <- factor(data$agecat, levels=1:3,
                      labels=c("<45", "45- <65", "65+"))
data$gender <- factor(data$gender, levels=1:2,
                      labels=c("Male", "Female"))
data$education <- factor(data$education, levels=1:3,
                         labels=c("Compulsory", "Secondary", "Tertiary"))
data$nat <- factor(data$nat, levels=0:1,
                      labels=c("Non-Swiss", "Swiss"))
data$partner <- factor(data$partner, levels=0:1,
                      labels=c("Living without partner", "Living with partner"))
data$work <- factor(data$work, levels=1:4,
                      labels=c("Employed", "Unemployed", "Retired", "Others"))

CreateTableOne(data=data %>%
                 select("agecat", "gender", "education", "nat",
                        "partner", "work", "lregion"))
```

```
##
##                                  Overall
##   n                              2026
##   agecat (%)
##      <45                         1018 (50.2)
##      45- <65                      704 (34.7)
##      65+                          304 (15.0)
##   gender = Female (%)             984 (48.6)
##   education (%)
##      Compulsory                   147 ( 7.3)
##      Secondary                    966 (47.9)
##      Tertiary                     903 (44.8)
##   nat = Swiss (%)                1834 (90.7)
##   partner = Living with partner (%) 1421 (70.1)
##   work (%)
##      Employed                    1436 (70.9)
##      Unemployed                    58 ( 2.9)
##      Retired                      299 (14.8)
##      Others                       233 (11.5)
##   lregion (%)
##      German/Romansh              1292 (63.8)
##      French                       437 (21.6)
##      Italian                      297 (14.7)
```

```r
xtable(output[1:30,], caption="Manuscript Table 2: Part 1", digits=c(0, 0, 3, 3, 3, 0))
```

| | var | val | lci | uci | n |
|---|---|---|---|---|---|
| 1 | unempl_prior | 0.025 | 0.019 | 0.035 | 1494 |
| 2 | unempl_covid | 0.013 | 0.009 | 0.021 | 1494 |
| 3 | wb1_cat | 0.933 | 0.921 | 0.943 | 2025 |
| 4 | wb2_cat1 | 0.858 | 0.842 | 0.873 | 2026 |
| 5 | wb3_cat3 | 0.414 | 0.393 | 0.436 | 2025 |
| 6 | ph1_cat1 | 0.881 | 0.866 | 0.894 | 2025 |
| 7 | ph2_1_cat1 | 0.043 | 0.035 | 0.053 | 2024 |
| 8 | ph2_2_cat1 | 0.052 | 0.043 | 0.063 | 2023 |
| 9 | ph2_3_cat1 | 0.013 | 0.009 | 0.019 | 2023 |
| 10 | ph2_4_cat1 | 0.016 | 0.012 | 0.023 | 2024 |
| 11 | ph2_5_cat1 | 0.070 | 0.060 | 0.082 | 2025 |
| 12 | ph2_6_cat1 | 0.037 | 0.029 | 0.046 | 2022 |
| 13 | ph2_7_cat1 | 0.006 | 0.004 | 0.011 | 2022 |
| 14 | ph2_8_cat1 | 0.008 | 0.005 | 0.013 | 2024 |
| 15 | ph2_9_cat1 | 0.005 | 0.003 | 0.010 | 2022 |
| 16 | ph2_10_cat1 | 0.056 | 0.047 | 0.067 | 2021 |
| 17 | ph2_11_cat1 | 0.011 | 0.008 | 0.017 | 2022 |
| 18 | ph2_12_cat1 | 0.006 | 0.003 | 0.010 | 2023 |
| 19 | ph2_1_cat3 | 0.604 | 0.582 | 0.625 | 2024 |
| 20 | ph2_2_cat3 | 0.560 | 0.538 | 0.581 | 2023 |
| 21 | ph2_3_cat3 | 0.819 | 0.801 | 0.835 | 2023 |
| 22 | ph2_4_cat3 | 0.794 | 0.776 | 0.812 | 2024 |
| 23 | ph2_5_cat3 | 0.522 | 0.500 | 0.544 | 2025 |
| 24 | ph2_6_cat3 | 0.657 | 0.636 | 0.677 | 2022 |
| 25 | ph2_7_cat3 | 0.896 | 0.882 | 0.909 | 2022 |
| 26 | ph2_8_cat3 | 0.875 | 0.860 | 0.889 | 2024 |
| 27 | ph2_9_cat3 | 0.967 | 0.959 | 0.974 | 2022 |
| 28 | ph2_10_cat3 | 0.602 | 0.581 | 0.623 | 2021 |
| 29 | ph2_11_cat3 | 0.753 | 0.733 | 0.771 | 2022 |
| 30 | ph2_12_cat3 | 0.904 | 0.890 | 0.916 | 2023 |

Table 1: Manuscript Table 2: Part 1

```r
xtable(output[31:70,], caption="Manuscript Table 2: Part 2", digits=c(0, 0, 3, 3, 3, 0))
```

| | var | val | lci | uci | n |
|---|---|---|---|---|---|
| 1 | ph2__1__cat2 | 0.353 | 0.333 | 0.374 | 2024 |
| 2 | ph2__2__cat2 | 0.388 | 0.367 | 0.409 | 2023 |
| 3 | ph2__3__cat2 | 0.169 | 0.153 | 0.186 | 2023 |
| 4 | ph2__4__cat2 | 0.189 | 0.173 | 0.207 | 2024 |
| 5 | ph2__5__cat2 | 0.408 | 0.387 | 0.429 | 2025 |
| 6 | ph2__6__cat2 | 0.307 | 0.287 | 0.327 | 2022 |
| 7 | ph2__7__cat2 | 0.097 | 0.085 | 0.111 | 2022 |
| 8 | ph2__8__cat2 | 0.117 | 0.103 | 0.131 | 2024 |
| 9 | ph2__9__cat2 | 0.027 | 0.021 | 0.035 | 2022 |
| 10 | ph2__10__cat2 | 0.342 | 0.322 | 0.363 | 2021 |
| 11 | ph2__11__cat2 | 0.236 | 0.218 | 0.255 | 2022 |
| 12 | ph2__12__cat2 | 0.090 | 0.078 | 0.103 | 2023 |
| 13 | ms2__1__cat1 | 0.065 | 0.055 | 0.077 | 2022 |
| 14 | ms2__1__cat3 | 0.353 | 0.333 | 0.374 | 2022 |
| 15 | ms2__1__cat2 | 0.582 | 0.560 | 0.603 | 2022 |
| 16 | ms2__2__cat1 | 0.030 | 0.024 | 0.039 | 2023 |
| 17 | ms2__2__cat3 | 0.613 | 0.592 | 0.634 | 2023 |
| 18 | ms2__2__cat2 | 0.357 | 0.336 | 0.378 | 2023 |
| 19 | ms2__3__cat1 | 0.083 | 0.072 | 0.096 | 2023 |
| 20 | ms2__3__cat3 | 0.097 | 0.085 | 0.111 | 2023 |
| 21 | ms2__3__cat2 | 0.820 | 0.802 | 0.836 | 2023 |
| 22 | ms2__4__cat1 | 0.056 | 0.047 | 0.067 | 2023 |
| 23 | ms2__4__cat3 | 0.418 | 0.396 | 0.439 | 2023 |
| 24 | ms2__4__cat2 | 0.526 | 0.504 | 0.548 | 2023 |
| 25 | ms2__5__cat1 | 0.077 | 0.066 | 0.090 | 2024 |
| 26 | ms2__5__cat3 | 0.071 | 0.060 | 0.083 | 2024 |
| 27 | ms2__5__cat2 | 0.852 | 0.836 | 0.867 | 2024 |
| 28 | ms2__6__cat1 | 0.165 | 0.149 | 0.181 | 2022 |
| 29 | ms2__6__cat3 | 0.037 | 0.030 | 0.046 | 2022 |
| 30 | ms2__6__cat2 | 0.798 | 0.780 | 0.815 | 2022 |
| 31 | ms2__7__cat1 | 0.090 | 0.079 | 0.104 | 2025 |
| 32 | ms2__7__cat3 | 0.266 | 0.247 | 0.286 | 2025 |
| 33 | ms2__7__cat2 | 0.643 | 0.622 | 0.664 | 2025 |
| 34 | ss3__catx | 0.098 | 0.086 | 0.111 | 2026 |
| 35 | hs1__1__cat | 0.156 | 0.141 | 0.173 | 2025 |
| 36 | hs2__1__cat | 0.212 | 0.195 | 0.230 | 2026 |
| 37 | wc1__cat | 0.105 | 0.090 | 0.121 | 1435 |
| 38 | wc2__cat | 0.543 | 0.517 | 0.568 | 1435 |
| 39 | hb1__cat | 0.036 | 0.028 | 0.045 | 2025 |
| 40 | hb7__cat | 0.185 | 0.169 | 0.203 | 2025 |

Table 2: Manuscript Table 2: Part 2
